# Quadruple fortification of salt for the delivery of iron, iodine, folic acid, and vitamin B_12_ to vulnerable populations

**DOI:** 10.1016/j.jfoodeng.2021.110525

**Published:** 2021-07

**Authors:** Oluwasegun Modupe, Levente L. Diosady

**Affiliations:** Department of Chemical Engineering and Applied Chemistry, University of Toronto, Canada

## Abstract

A process for simultaneous delivery of iron, iodine, folic acid, and vitamin B_12_ through salt as a potential and holistic approach to ameliorate anaemia and reduce maternal and infant mortality is presented. Two approaches for adding folic acid and B_12_ to salt during double fortification with iron and iodine were investigated. Attempts to add both micronutrients through the iodine spray solution were unsuccessful. Hence, folic acid was added through a stabilized iodine solution, and B_12_ was added through the iron premix. Four approaches used to incorporate B_12_ into the iron premix were investigated: (1) co-extruding B_12_ with iron, (2) spraying B_12_ on the surface of the iron extrudate, (3) adding B_12_ to the colour masking agent, and (4) adding B_12_ to the outer coating. Of these approaches, coextrusion (1) was the best, based on the ease of production and stability of fortificants. The salt formulated with the solid iron-B_12_ premix and sprayed iodine and folic acid solution contained 1000 ppm iron, 50 ppm iodine, 25 ppm folic acid, and 0.25 ppm B_12_. Over 98% of B_12_, 93% folic acid, and 94% iodine were retained after 6-month storage in the best formulation. This technology can simultaneously deliver iron, iodine, folic acid, and vitamin B_12_ in a safe and stable salt enabling public health measures for improved health at a minimal additional cost.

## Highlights

• The process developed simultaneously delivers iron, iodine, folic acid, and vitamin B_12_ through salt.
• The chemistry of interaction among the micronutrients guided the process development.
• Iron and vitamin B_12_ were added as encapsulated particles (premix), while iodine and folic acid were added as a solution.
• Vitamin B_12_ and folic acid had different pH range requirements; hence, they were incompatible in solution.
• Of the four iron-B_12_ premix designs evaluated, the coextrusion of iron and vitamin B_12_ was the best design for the process.
• The additional cost of adding these micronutrients to salt is about $0.30 per year per person; hence, very cost effective.

## 1 Introduction

Adding iron to salt had significantly reduced anaemia in major efficacy studies (Horton et al., 2011/10; Ramírez-Luzuriaga et al., 2018). Like iron, vitamin B_9_ (folic acid) and vitamin B_12_ are micronutrients that directly impact the synthesis of red blood cells. Therefore, their deficiencies are a risk factor for anaemia. Accordingly, efforts to reduce the global prevalence of anaemia by fortifying salt with iron should be complemented by adding vitamin B_9_ and B_12_ to iron so that they are delivered simultaneously through iodized salt. Iron deficiency has adverse effects on human working capacity, motor, and mental development (Cappellini et al., 2017 ). Folic acid and Vitamin B_12_ deficiencies are risk factors for neural tube defects, neurological disorders, vascular disease, and cognitive impairment (Stabler, 2013; Williamset al., 2015). Therefore, quadruple fortification of salt is expected to positively impact health and quality of life in populations suffering from these micronutrient deficiencies.

Salt is one of the very few manufactured foods consumed by everyone, reaching populations that do not have access to or cannot afford other foods fortified with micronutrients. The range of salt intakes is limited by taste rather than economics, so the individual salt consumption in a population is predictable. Hence, the amount of micronutrients needed in salt is readily defined. Technology for adding iron to salt was developed to leverage the established process, equipment, and distribution channels widely available for iodized salt (Rao, 1994; Wegmüller et al., 2003; Oshinowo et al., 2004; Romita et al., 2011; Li et al., 2010). The technology developed at the Food Engineering Laboratory at the University of Toronto specified that iron should be added as an agglomerated and microencapsulated ferrous fumarate to iodized salt to produce Double Fortified Salt (DFS) (Romita et al., 2011; Li et al., 2010; Oshinowo et al., 2012). The process has been scaled up in India, with the DFS reaching about 60 million people in the recent test programs (Diosady et al., 2019).

The process for adding folic acid, the synthetic form of vitamin B_9_, to DFS was developed by McGee (2012) and Modupe et al. (Modupe et al., 2019). McGee (2012) added folic acid to a buffered iodine solution, which was sprayed on salt. However, the low concentration of folic acid and iodine (0.35% ^w^/_v_, each) required adding much more solution to salt to meet the targeted concentration of folic acid and iodine in salt. The high moisture content in the Triple Fortified Salt (2.9%) prepared by McGee (2012) accelerated iodine loss in the Triple Fortified Salt (TFS). Modupe et al. (Modupe et al., 2019) minimized iodine loss in the salt by increasing folic and iodine concentration in the spray solution to 1% and 2% ^w^/_v_, respectively. The moisture content of the salt was reduced from 2.9% to 0.06%.

As described by Modupe et al. (Modupe et al., 2019), the formulation of TFS entailed adding iron as a microencapsulated extruded ferrous fumarate (iron premix), while a solution containing 2% ^w^/_v_ iodine and 1% ^w^/_v_ folic acid was sprayed onto the salt. Li et al. (Li, 2009) and Yadava et al. (2012) described how the iron premix was made. The folic acid and iodine solution was formulated and adjusted to pH 9 with 0.1 M Na_2_CO_3,_ as described by Modupe et al. (Modupe et al., 2019). The resulting salt can deliver up to 50% of the Recommended Dietary Allowance (RDA) for iron, >200% of RDA for iodine, and 100% of RDA for folic acid.

Since vitamin B_12_ deficiency is also a risk factor for anaemia, and the metabolisms of folic acid and vitamin B_12_ are intertwined, it is desirable to add folic acid and vitamin B_12_ simultaneously. In the process for the triple fortification of salt (Modupe et al., 2019), there are two possible places where vitamin B_12_ could be added to salt – through the iodine and folic acid solution or the iron premix. This study determined the optimum technique for adding vitamin B_12_ and folic acid to Double Fortified Salt to ensure that over 70% of the micronutrients are retained after six months of storage while meeting at least 30% of the RDA of each of these micronutrients in the quadruple fortified salt (QFS). The marginal cost of producing QFS is low and can positively impact the health of low-income populations that are vulnerable to micronutrient deficiencies.

## 2 Materials and experimental methods

### 2.1 Materials

Refined salt (~400 μm diameter) was obtained from Sifto (Canada) Corp. Potassium iodate (iodine source), potassium iodide, acetonitrile, methanol, sulfuric acid, sodium thiosulfate solution, sodium citrate, sodium ascorbate, sodium erythorbate, and sodium carbonate were obtained from Sigma–Aldrich Chem (Oakville, Ontario, Canada). Ferrous fumarate (iron) was obtained from Dr. Paul Lohmann Chemicals (Emmerthal, Germany). Soy stearin and cyanocobalamin (vitamin B_12_) were obtained from JVS Foods Pvt Ltd (India). Folic acid was obtained from Bulk Pharmaceuticals Inc. (Toronto, Ontario, Canada). Hydroxypropyl methylcellulose (HPMC), Crisco Shortening, semolina, and titanium (IV) oxide were obtained from Dow Chemicals Co. (Midland, MI, United States), J.M. Smucker Co. (USA), Walmart (Toronto, Canada), and ACROS Organics (USA) respectively.

All chemicals used for the fortification of salt were food-grade, while those used for analysis were ACS grade.

### 2.2 Experimental methods

#### 2.2.1 Formulation and storage of spray solution

Initially, highly concentrated solutions were made, containing 3% iodine (I_2_), 2% folic acid (FA), 0.015% vitamin B_12_ (B_12_), 1% erythorbate, 1% ascorbate and or 1% citrate. Six solutions were formulated with 0.2 M sodium carbonate, to maintain pH near 9, thus:

(a) 3% I_2_ + 2% FA,
(b) 3% I_2_ + 2% FA + 0.015% B_12_,
(c) 3% I_2_ + 2% FA + 0.015% B_12_ + 1% erythorbate,
(d) 3% I_2_ + 2% FA + 0.015% B_12_ + 1% ascorbate,
(e) 3% I_2_ + 2% FA + 0.015% B_12_ + 1% citrate, and
(f) 0.015% vitamin B_12_

Due to precipitation in some of the solutions (a-d) after a few days of storage and the perceived impact of pH on the stability of vitamin B_12_ in the solutions (a-f), another set of solutions were formulated. This second set of four solutions, which only differed in pH, contained 0.01% vitamin B_12_, 1% folic acid, and 2% iodine. Sodium carbonate solution (0.1 M) was used to dissolve folic acid and adjust the solutions to pH 8–11. Finally, seven solutions containing folic acid, vitamin B_12_, iodine, sodium ascorbate, sodium citrate, and sodium carbonate were prepared as presented in Table 1. Based on the observed impact of high pH on B_12_ stability, the final set of solutions was adjusted to pH 9 or less. The stability of micronutrient in the solutions was monitored for 2 months at 25 °C, 35 °C, and 45 °C in the dark and under natural light in the laboratory at 25 °C. For the samples exposed to light, the solutions were stored in transparent scintillation vials and kept on a laboratory bench. The stability of micronutrients in the solutions was expressed as a percentage of the micronutrient concentrations in the freshly prepared samples.

**Table 1:** Formulation design for spray solutions.

Spray Solutions	Constituents						pH
	Iodine (%)	Folic acid (%)	Vitamin B_12_ (%)	Ascorbate (%)	Citrate (%)	Na_2_CO_3_ (%)	
1	0	0	0.01	1.00	0	0	2.80
2	2.00	1.00	0.01	0	0	0.74	9.00
3	2.00	1.00	0	0	0	0.74	9.00
4	2.00	0	0.01	0	0	0	6.90
5	0	0	0.01	0	0	0	6.50
6	2.00	1.00	0.01	0	1.00	ND	9.00
7	2.00	1.00	0.01	0	0	ND	8.00

*ND= The amount of sodium carbonate used was not determined; in these cases, a 0.1 M Na_2_CO_3_ solution was used to adjust the solution's pH (Solution 6 &7).

#### 2.2.2 Production of iron and Iron-B_12_ premix

**Fig. 1:**
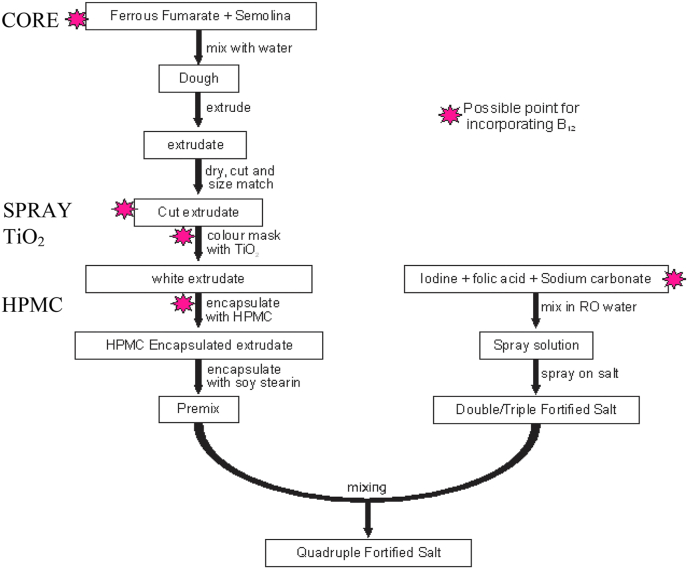
Process Flow diagram for Quadruple Fortified Salt (QFS) formulations (Modupe et al., 2021).

The micronutrients must be stable in solution for at least two months since many local iodized salt manufacturing plants store their iodine solution for weeks to reduce preparation costs. Since vitamin B_12_ was unstable in folic acid-containing solutions, it was incorporated into the solid iron premix instead. The process described by Li et al. (Li, 2009) and Yadava et al. (2012) for formulating the premix was modified (as illustrated in Fig. 1). For making the iron premix, semolina (200 g), ferrous fumarate (800 g), melted vegetable shortening (25 g), and water (120–150 mL) were thoroughly mixed with a KitchenAid mixer. The iron-containing dough was extruded with a La Monferrina P12 pasta extruder. The extrudate was cut and screened to obtain 500–600 μm cylindrical particles, which were colour masked with 25% TiO_2,_ and coated, first, with 5% HPMC using a fluidized bed, then with 5% soy stearin, using a pan coater. For the iron-B_12_ premix, four approaches were adopted: (1) CORE: B_12_ was co-extruded with iron, (2) SPRAY: a solution of B_12_ was sprayed onto iron extrudate before colour masking, (3) TiO_2_: B_12_ was added to the TiO_2_ used for colour masking, and (4) HPMC: B_12_ was added to HPMC used for the initial coating (See Fig. 1). The TiO_2_ approach was not feasible; hence, only the other three were subsequently tested. The stability of B_12_ in the premixes was evaluated over six months. The stability was expressed as the percentage of the original B_12_ retained in the samples. The pH of 0.1 g premix samples dissolved in 100 mL RO water was determined using a WR Scientific Model 8000 pH meter.

#### 2.2.3 Formulation of fortified salt

Since B_12_ was not stable in the folic acid-containing iodate solution, a solution containing only 1% folic acid and 2% iodine (3.37% potassium iodate) was sprayed on salt in most of the experiments, while iron and B_12_ were admixed to salt as a solid. However, a freshly prepared solution containing 1% folic acid, 2% iodine, and 0.01% B_12_ (solution 2) was also tested for comparison. The solution (5 mL) was sprayed on salt (2 kg) inside a ribbon blender and thoroughly mixed for 20 min. The salt samples were dried overnight. The dried salt was returned to the ribbon blender and mixed with the premix for 2 min. Four quadruple (QFS) and four triple fortified salts (TFS*) were formulated with target concentrations of 1000 ppm iron, 50 ppm iodine, 25 ppm folic acid, and 0.25 ppm B_12_ as presented in Table 2.

**Table 2:** Formulation design for QFS.

Salt Samples	Constituents (ppm)				Source of Vit. B_12_
	Iron	Folic acid	Iodine	Vit. B_12_	
QFS Core	1000	25	50	0.25	CORE Fe–B_12_ Premix
QFS Spray	1000	25	50	0.25	SPRAY Fe–B_12_ Premix
QFS HPMC	1000	25	50	0.25	HPMC Fe–B_12_ Premix
QFS (iron premix)	1000	25	50	0.25	Solution 2
TFS* Core (No FA)	1000	0	50	0.25	CORE Fe–B_12_ Premix
TFS* Core (No I)	1000	25	0	0.25	CORE Fe–B_12_ Premix
TFS* HPMC (No FA)	1000	0	50	0.25	HPMC Fe–B_12_ Premix
TFS* HPMC (No I)	1000	25	0	0.25	HPMC Fe–B_12_ Premix

In all cases except where the iron premix was sprayed with Solution 2 containing iodine, folic acid, and vitamin B_12_, a premix containing iron and vitamin B_12_ was used to formulate the fortified salt. The three iron-vitamin B_12_ premix samples (CORE, SPRAY, and HPMC) described above were used. TFS* samples contained iron, iodine, and B_12_ or folic acid.

### 2.3 Handling and storage of the fortified salt samples

The fortified salts were divided by sample splitter glassware into three portions of approximately 666 g each. The salt samples were packaged in Ziplock™ freezer bags and stored at 25 °C, 35 °C, and 45 °C, 60–70 %RH. Since the B_12_ was very stable in the premix, only folic acid and iodine stability were monitored in the salt. The micronutrients in the salt were quantified when they were freshly formulated and after six months.

#### 2.3.1 Iron in vitro bioavailability approximation

First, the total iron in the premix was determined. The premix (100 mg) was added into digestion vials containing 15 mL 3:1 mixture of concentrated hydrochloric acid and nitric acid. The sample was digested for 2 h using a microwave digester (ETHOS EZ, Milestone Inc.). The resulting solution was made up to 50 mL in a volumetric flask with 5% ^v^/_v_ nitric acid, then further diluted at a ratio of 1:10 with 5% ^v^/_v_ nitric acid. The sample's iron content was analyzed using an inductively coupled plasma optical emission spectrometer (ICP-OES, Agilent Dual View 720).

The in vitro bioavailability was approximated by measuring the fraction of iron dissolved at stomach pH. In the test, premix (100 mg) was added into a 500 mL Erlenmeyer flask that contained 250 mL 0.1 M HCl solution. The flask was placed in a Cole-Parmer StableTemp Water bath (EW-14575-12) coupled with Cole-Parmer Polyscience Dual Action Shaker, set at 37 °C, 160 rpm for 2 h. Some of the solution (1 mL) was withdrawn from the flask at 30-min intervals for 2 h. The withdrawn solution was mixed with 9 mL 5% ^v^/_v_ nitric acid, then filtered with a 45 μm syringe filter. The concentration of the iron in the filtrate was measured using the ICP-OES. The amount was presented as a percentage of the total amount of iron in the premix.

### 2.4 Folic acid analysis

The method described by Modupe et al. (2020) was used to quantify folic acid in the salt and spray solution. For the spray solution, samples were diluted with 0.1 M Na_2_CO_3_ at a ratio of 1:1000_,_ and the absorbance of the resulting solution was read at 285 nm. For the salt samples, 5 g salt was dissolved in 0.1 M Na_2_CO_3_ (10 mL) inside a 50 mL falcon tube. The solution was mixed with a vortex mixer for 2 min and filtered with a 0.45 μm syringe filter. The absorbance of the filtrate was immediately read at 285 nm.

### 2.5 Iodine analysis

Method 33.149, described by the Association of Official Analytical Chemists (AOAC), was used for quantifying iodine in salt and spray solutions (Association of Official Analytical Chemists, 1984). In this method, iodate was reduced to iodine and titrated with sodium thiosulfate using starch as an indicator.

#### 2.5.1 B_12_ analysis

The spray solution was diluted with distilled water at a ratio of 1:10. The reconstituted solution was filtered with a 0.45 μm syringe filter. The B_12_ in the filtrate was quantified with UHPLC-MS.

Premix was pulverized with a mortar and pestle, and 5 g of the pulverized premix was weighed into a 50 mL falcon tube; 10 mL of RO water was added and mixed with a vortex mixer for 2 min. It was centrifuged for 2 min. The supernatant was filtered with a 0.45 μm filter. The B_12_ in the filtrate was quantified using UHPLC-MS.

#### 2.5.2 Cost analysis

The reported process is based on traditional techniques for making iodized salt. Converting iodized salt to QFS requires only a piece of mixing equipment with proven performance in making Double Fortified Salt. In addition to this capital cost, the added micronutrients is another major cost of QFS preparation. The material costs were obtained from vendor websites. Manufacturing cost was approximated as wages of personnel in a salt manufacturing plant in India, factoring in the expected time required for the process (Diosady et al., 2019).

#### 2.5.3 Statistical analysis

At least four replicates were used in all the experiments. For each micronutrient and independent replicate, salt sold in a Ziplock bag was mixed before the contents were sampled. The results are expressed as a mean ± SD. The data were subjected to one-way ANOVA using SPSS software, and the differences between means were considered significant at P < 0.05. Only differences with the significance of P < 0.05 are mentioned in all subsequent discussions.

## 3 Results

**Fig. 2:**
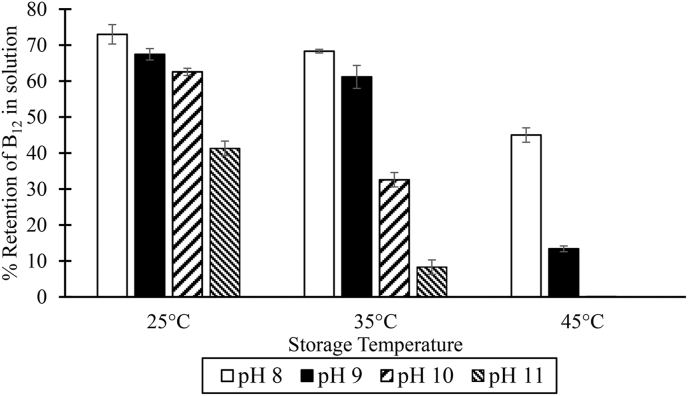
Impact of pH and Temperature on the Stability of Vitamin B_12_ in Iodine and Folic Acid Solution after One Month of Storage.

Initially, precipitation was observed after a few days of storage in solutions (a-d) that contained 0.015% vitamin B_12_, folic acid (2%), and iodine (3%). Of the three antioxidants (erythorbate, citrate, or ascorbate) added, only the citrate helped keep the micronutrients in solution (Solution e). All vitamin B_12_ was lost after one month of storage. The total loss of vitamin B_12_ was attributed to the solution's high pH. The spray solution's pH significantly impacted the stability of vitamin B_12_ in the tested range of pH 8 to 11. Vitamin B_12_ stability decreased with increased pH (Fig. 2).

The retention of B_12_ in the solution (0.01% B_12_ + 2% Iodine + 1% Folic acid) was expressed as a percentage of B_12_ in fresh samples. The pH of the solutions was adjusted with 0.1 M Na_2_CO_3_.

Vitamin B_12_ was not stable in any solution that contained folic acid (Solutions 4 and 5). Almost all of the vitamin B_12_ in the spray solution at pH 9 was lost after two months of storage at 45 °C (Fig. 3). Even at a lower pH value, more than 50% of the added B_12_ was lost. Neither citrate nor ascorbic acid improved the stability of vitamin B_12_ in the solution (Fig. 3). However, B_12_ was relatively stable in solutions that contained only B_12_ and iodine without pH adjustment. When exposed to natural light, ascorbic acid accelerated the photolytic degradation of vitamin B_12_ in the solution. Solution 1, containing ascorbic acid, turned from pink to yellow after one month of storage when exposed to light.

**Figue 3:**
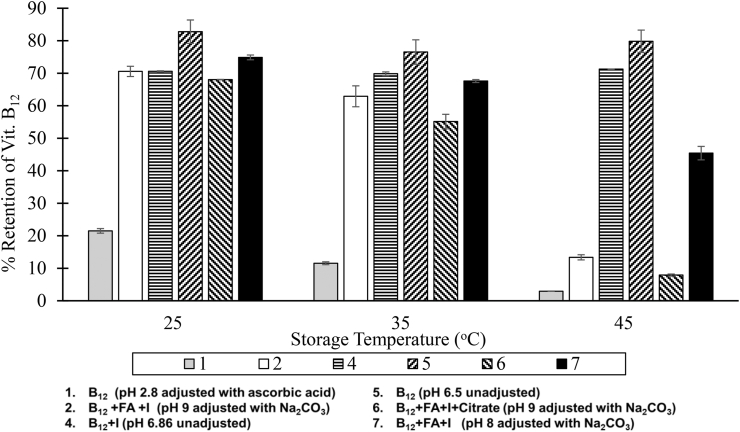
Impact of pH on the Stability of Vitamin B_12_ in Solution after Two Months of Storage.

The solution pH was adjusted with 1% sodium citrate, 1% sodium ascorbate, or 0.74% sodium carbonate.

Given the failed attempts of adding vitamin B_12_ to the iodine and folic acid spray solution, vitamin B_12_ was incorporated into the iron premix so that the solid premix contained iron and B_12_. There were four approaches attempted for adding B_12_ to the iron premix. Of these, co-extruding B_12_ and iron (CORE Fe–B_12_ premix) was the most technically successful. Spraying B_12_ on the premix's surface (SPRAY Fe–B_12_ premix) required a significant amount of water to make the B_12_ uniformly distributed on the premix, which increased the moisture content of the premix. This resulted in a need for another drying stage for making the premix.

Adding B_12_ to the HMPC resulted in pink premix particles that contrast sharply with the white salt (HPMC Fe–B_12_ Premix). Also, adding B_12_ through the colour masking agent (TiO_2_) was not technically feasible, as most of the B_12_ was lost with TiO_2_ in the final coating unit operation where some titanium dioxide and B_12_ held loosely on the iron extrudate surface were blown away by the compressed air required to fluidize the particles during coating in a fluidized bed coater (TiO_2_ Fe–B_12_ Premix). Since we could not accurately predict TiO_2_ and B_12_ were lost during this coating step, this approach was not used further. Vitamin B_12_ was very stable in the other three premix samples. In each, over 97% of the B_12_ was retained after six months of storage. Of these three approaches, technically, co-extruding iron and B_12_ (CORE Fe–B_12_) was the simplest. The pH of the premix (4.7–5.0) was optimal for the stability of B_12_.

In formulating these QFS samples, a solution of iodine and folic acid was sprayed on salt while three Fe–B_12_ premix samples (CORE Fe–B12, SPRAY Fe–B12, and HPMC Fe–B12 premix) were used. After six months of storage, the amount of folic acid and iodine in the salt samples was expressed as a percentage of the micronutrients retained from the freshly prepared QFS.

**Fig. 4:**
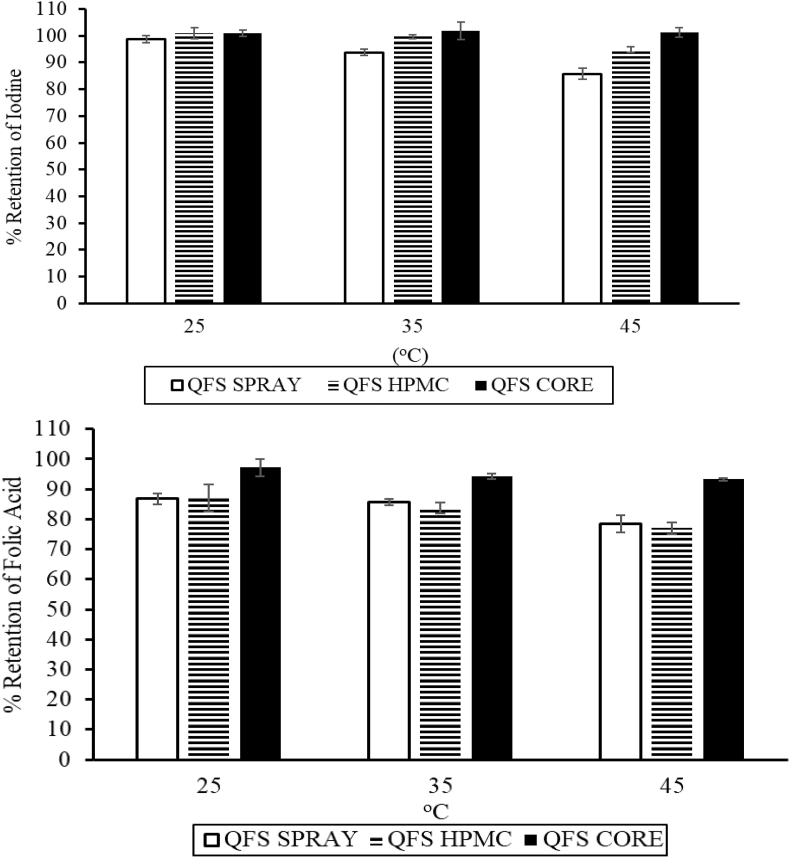
Stability of Iodine and Folic Acid in QFS Formulated with the different Premix Samples after 6 Months Storage.

**Fig. 5:**
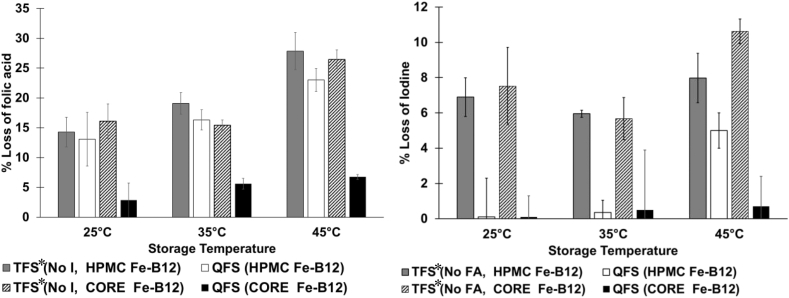
Impact of folic acid on iodine loss and iodine on folic acid loss in fortified salt after 6-month storage.

These three premix samples were used to formulate QFS that contained 1000 ppm iron, 50 ppm iodine, 25 ppm folic acid, and 0.25 ppm B_12_. In all the cases, 74–97% of folic acid and 85–100% of iodine were retained in the QFS after six months of storage at 25, 35, and 45 °C, 60–70% RH (Fig. 4, Fig. 5).

The point of addition of B_12_ did not impact the iodine stability at room temperature, but at 45 °C, iodine's stability was significantly higher when B_12_ was incorporated in the ferrous fumarate core. (Fig. 4). Incorporating the B_12_ in the core resulted in 6–15% higher iodine retention than in the other two B_12_ locations in the premix. A similar trend was observed for folic acid. Folic acid was significantly more stable in QFS formulated with the B_12_ in the core at all the temperatures. Folic acid retention was 11–15% higher when B_12_ was in the core rather than in other parts of the premix (Fig. 4). Folic acid and iodine were more stable in the QFS samples where B_12_ was in premix than when sprayed as a solution (along with folic acid and iodine). Even without considering the folic acid-B_12_ spray solution's long-term instability, more iodine, and folic acid (10–20%) were lost in the sample prepared with the combined spray (Fig. 4). This loss may have been due to the interactions between vitamin B_12,_ iodine, and folic acid in the salt.

Four types of Triple Fortified Salt (TFS*) were formulated with a folic acid solution (No I), an iodine solution (No FA), and two premix samples (HPMC Fe–B_12_ or CORE Fe–B_12_). The retention of folic acid or iodine in the TFS was compared with iodine or folic acid retention in two types of QFS. The QFS samples were formulated with folic acid and iodine solution and two premix samples (HPMC Fe–B_12_ or CORE Fe–B_12_).

Folic acid and iodine mutually stabilized each other in QFS (Fig. 5), as the iodine and folic acid in QFS samples, which had both iodine and folic acid in contact, were more stable than in samples that did not have either iodine or folic acid. The same trend was reported by McGee et al. (2017). More importantly, these results showed that technology could be used to simultaneously deliver iodine, iron, and B_12_ in case of a population with sufficient folic acid deficient in vitamin B_12_. These results showed that the targeted stability of micronutrients in the QFS (70%) was met. However, the salt was yellow because folic acid was sprayed directly on the salt.

**Fig. 6:**
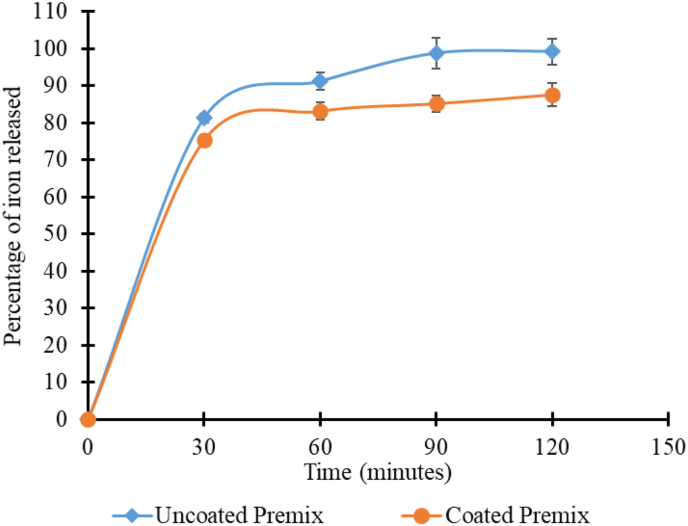
Iron release profile from uncoated and coated premix samples.

The premix was designed to disintegrate during most cooking methods, releasing iron and B_12_ in a bioaccessible form. Still, iron in vitro bioavailability was evaluated for the premix for cases where the intact premix is ingested. Over 80% of the iron in the premix was released into the 0.1 N HCl (similar to gastric juice concentration) after 2 h (Fig. 6). Since 0.1 N HCl could digest the coat and disintegrate the premix, vitamin B_12_ in the premix would be bioavailable also. The result is similar to iron bioavailability reported for iron premix with a similar coating (Li et al., 2009).

The results were represented as a percentage of the total in the premix sample.

Although almost everyone could benefit from fortified salt, the technology primarily targets poor rural women, who have no access to fortified processed food products, such as flour. Hence, the cost implication for adding the four micronutrients through this technology was examined. The additional material cost for adding the four micronutrients is 8.5 cents/kg salt. Based on the assumption that on average, 10 g of salt is consumed per person per day in the target populations, the additional cost of adding iron, iodine, folic acid, and vitamin B_12_ to salt is less than 31 cents per person per year (Table 3). This cost estimate is very close to that projected at the Pilot Plant.

**Table 3:** Cost analysis for the quadruple fortification of salt.

Constituents of QFS	USD/kg	Amount needed/kg of salt	US'/kg salt
Ferrous fumarate	12.00	2.91 g	3.49
Semolina	1.80	0.73 g	0.13
Fat	1.57	0.09 g	0.01
TiO_2_	22.00	0.91 g	2.00
Soy Stearin	2.00	0.18 g	0.04
HPMC	52.00	0.18 g	0.95
Sodium Carbonate	9.70	0.02 g	0.02
Potassium Iodate	6.00	0.08 g	0.05
Folic Acid	90.00	0.03 g	0.23
Cyanocobalamin	5000.00	250 mg	0.13
Personnel cost for Premix	3.00	5 g premix	1.50
Total cost/kg of salt			8.54
Cost per person/year			31.12

The cost analysis was based on material and personnel costs. Most of the material prices were obtained from the Alibaba website, while the personnel cost was based on the wages of personnel in a salt manufacturing plant in India (Modupe, 2020).

## 4 Discussion

The simplest technique for making Quadruple Fortified Salt would be to add folic acid, B_12,_ and iodine through a sprayed solution and admixing iron as an encapsulated solid premix. However, folic acid requires a high pH for solubility, and vitamin B_12_ is unstable in alkaline solution. Even at pH 8, more than 30% of the added vitamin B_12_ was lost in two months, missing the minimum stability target of at least 70% retention. Attempts to stabilize vitamin B_12_ in the solutions with antioxidants (citrate, erythorbate, and ascorbate) proved ineffective. Hence, formulating QFS by spraying a solution containing iodine, folic acid, and B_12_ is not feasible.

Ascorbate concentration (AH_2_) and pH were the most significant factors that negatively affected the stability of B_12_ in the solutions. Ascorbate promoted the degradation of B_12_ in the solution. Ahmad et al. (Ahmad et al., 2017/01), (Ahmadet al.eng, 2014) described the degradation product as hydroxocobalamin, which is further rapidly degraded to products that do not elicit the vitamin functions. The degradation of cyanocobalamin to hydroxocobalamin involves the loss of the CN-group. The degradation is a reductive decyanation reaction that occurs slowly in the presence of light, but in synergy with ascorbate, the reaction rate increased (Ahmadet al.eng, 2014). The reductive potential of the ascorbate is responsible for this.

Given that ascorbate pKa is 4.2 (Du et al., 2012), it is predominantly in its conjugate acid form in the solution (pH 2.8), which has high reductive potential to drive the decyanation. The spray solution that contained ascorbates was discoloured when exposed to light for one month. The colour change implies that the central cobalt was reduced from Co^3+^ to Co^2+^, and the corrin ring was oxidatively cleaved (Frost et al., 1952). Since Co^2+^ moiety in vitamin B_12_ can be easily oxidized to Co^3+^ (Fedosov et al., 2011) and the loss of vitamin function is through oxidation, the oxidative cleavage poses more significant damage to the stability of vitamin B_12_ than reductive decyanation. The mechanisms are summarized in Equation (1).

(1) $$\left[Co^{3+}CN\right]\overset{\;hv/AH_{2}\;}{\to }\left[Co^{3+}OH\right]+CN^{-}\overset{\;hv/AH^{-}}{\to }\left[Co^{2+}OH\right]\overset{O_{2}}{\to }\;Corrin\;ring\;cleavage\;products$$

The incompatibility of folic acid and vitamin B_12_ in the solutions suggested removing one of the micronutrients from the solution. The comparatively higher stability of folic acid (91% vs. 70% for B_12_) in the solution lead to the decision to move vitamin B_12_ from the spray solution to the solid iron premix. Of the four techniques evaluated for making B_12_ containing premix, co-extruding iron and vitamin B_12_ was the best. It is technically the least challenging of the four techniques. The technique physically separates the incompatible fortificants, and the coating protects B_12_ from photodegradation and may also facilitate the acceptability of the QFS as it masks the pink colour of the vitamin B_12_. The micronutrients were very stable in the QFS, with more than 90% of all the added micronutrients retained during 6-months storage at the highest expected local temperatures, 45 °C.

The consumer acceptance of the salt's yellow colour due to folic acid may require educating the target populations about the health benefits of QFS. The existing infrastructure for DFS production can be readily adapted for making QFS. Folic acid can be added to the iodine solution and sprayed with the existing iodization equipment, while B_12_ can be incorporated within the iron premix and added without a change in processing equipment or operation. Hence, no capital cost should be incurred in upgrading a salt mill from DFS to QFS. Based on the cost of added micronutrients and additional operating costs, providing QFS will cost ~30 cents per person per year more than the currently available iodized salt making this process a cost-effective approach for delivering micronutrients to vulnerable populations.

## 5 Summary

The technique described for making fortified salt can simultaneously deliver iron, iodine, folic acid, and vitamin B_12_ to vulnerable populations and has an excellent prospect for reducing or preventing aneamia and reducing maternal and infant mortality in populations with a high prevalence of these micronutrients deficiencies. As incorporating both folic acid and B_12_ into the iodine spray solution was unsuccessful, B_12_ was incorporated into the iron premix. The coextrusion of iron and vitamin B_12_ results in a stable premix. The QFS can readily supply iron (50% RDA), iodine (200% RDA), folic acid (100% RDA), and B_12_ (100% RDA) based on 10 g salt consumption per day. In the premix, over 98% of B_12_ was retained after six months of storage, while in the QFS, 93% folic acid and 94% iodine were retained after six months of storage even at 45 °C, 60–70% RH. Existing facilities for making DFS can be readily adapted to making QFS without any change in equipment. Thus, it presents a cost-effective approach for delivering multiple micronutrients to vulnerable populations. The process is ready for scale-up through pilot and full-scale tests.

Further work may be needed to mask the yellow colour impacted to salt by folic acid if it impedes consumer acceptability in field trials.

## Author contributions

Oluwasegun Modupe carried out the experiments and prepared this manuscript. Diosady Levente was the Principal Investigator who initiated and supervised the study and edited the final manuscript.

## Funding

This research was funded by 10.13039/501100004828Grand Challenges Canada, the 10.13039/100000865Bill & Melinda Gates Foundation, (OPP1151531), and a Nigerian State Scholarship (Petroleum Technology Development Fund Overseas Scholarship).

## Declaration of competing interest

There is no conflict of interest to declare.
